# A Tumor-Targeted Replicating Oncolytic Adenovirus Ad-TD-nsIL12 as a Promising Therapeutic Agent for Human Esophageal Squamous Cell Carcinoma

**DOI:** 10.3390/cells9112438

**Published:** 2020-11-10

**Authors:** Zifang Zhang, Chunyang Zhang, Jinxin Miao, Zhizhong Wang, Zhimin Wang, Zhenguo Cheng, Pengju Wang, Louisa S. Chard Dunmall, Nicholas R. Lemoine, Yaohe Wang

**Affiliations:** 1 Sino-British Research Centre for Molecular Oncology, National Centre for International Research in Cell and Gene Therapy, School of Basic Medical Sciences, Academy of Medical Sciences, Zhengzhou University, Zhengzhou 450052, China; zifangzhang@zzu.edu.cn (Z.Z.); jinxin.miao@aggiemail.usu.edu (J.M.); wzhizhong@hotmail.com (Z.W.); wangzhimin1975@hotmail.com (Z.W.); chengzhenguo@zzu.edu.cn (Z.C.); wangpengju@zzu.edu.cn (P.W.); 2 Department of Surgical Sciences, The First Affiliated Hospital of Zhengzhou University, Zhengzhou 450052, China; zcy198200@163.com; 3 Department of Science and Technology, Henan University of Chinese Medicine, Zhengzhou 450046, China; 4 Centre for Biomarkers & Biotherapeutics, Barts Cancer Institute, Queen Mary University of London, London EC1M6BQ, UK; l.chard@qmul.ac.uk

**Keywords:** Ad-TD-nsIL12, antitumour efficacy, ESCC, oncolytic adenovirus, Syrian hamster model

## Abstract

Esophageal squamous cell carcinoma (ESCC) is one of the most lethal cancers in China and existing therapies have been unable to significantly improve prognosis. Oncolytic adenoviruses (OAds) are novel promising anti-tumor drugs and have been evaluated in several cancers including ESCC. However, the antitumour efficacy of the first generation OAds (H101) as single agent is limited. Therefore, more effective OAds are needed. Our previous studies demonstrated that the novel oncolytic adenovirus Ad-TD-nsIL12 (human adenovirus type 5 with *E1ACR2*, *E1B19K*, *E3gp19K*-triple deletions)harboring human non-secretory IL-12 had significant anti-tumor effect, with no toxicity, in a Syrian hamster pancreatic cancer model. In this study, we evaluated the anti-tumor effect of Ad-TD-nsIL12 in human ESCC. The cytotoxicity of Ad-TD-nsIL12, H101 and cisplatin were investigated in two newly established patient-derived tumor cells (PDCs) and a panel of ESCC cell lines in vitro. A novel adenovirus-permissive, immune-deficient Syrian hamster model of PDCs subcutaneous xenograft was established for in vivo analysis of efficacy. The results showed that Ad-TD-nsIL12 was more cytotixic to and replicated more effectively in human ESCC cell lines than H101. Compared with cisplatin and H101, Ad-TD-nsIL12 could significantly inhibit tumor growth and tumor angiogenesis as well as enhance survival rate of animals with no side effects. These findings suggest that Ad-TD-nsIL12 has superior anti-tumor potency against human ESCC with a good safety profile.

## 1. Introduction

Esophageal carcinoma (EA) is one of the most malignant gastrointestinal tumors. According to the pathological types, EA can be divided into esophageal squamous cell carcinoma (ESCC) and esophageal adenocarcinoma (EAC). In China, ESCC is the most common type and has become the fourth most lethal cancer [1,2]. Despite the continuous application of new therapies in clinical practice, the five-year survival rate of patients is still less than 30% [3]. Therefore, new treatment methods are urgently needed.

Oncolytic viruses (OVs) have recently shown great promise for treatment of cancer and one OV-based drug has been approved by FDA to treat cancer [4,5]. OVs selectively infect and lyse tumor cells, secreting potent virions which can further infect nearby cancer cells and spread within tumor tissues. OV infection is also a potnet stimulator of host immune responses, inciting the development of robust antitumor immunity, which in addition to eliminating the primary tumour, can help prevent tumor metastasis and recurrence [6]. Oncolytic adenoviruses (OAds) are the most commonly used viruses in anti-cancer research because of their high safety, efficacy and easy genetic editability. H101 (*E1B-55K*/*E3B*-deleted adenovirus) was the first commercial OAd approved by the Chinese State Food and Drug Administration to treat nasopharyngeal carcinoma. It has also been evaluated for efficacy in several other cancers including lung and liver cancer [7,8,9]. Unfortunately, H101-related efficacy is poor, largely due to the deletion of the *E1B55K* and *E3B* genes, which dramatically reduce the replicative ability of virus and accelerate the clearance rate of virus from tumor tissue [10]. Combination of H101 with conventional chemotherapeutic drugs has been investigated to improve clinical efficacy, however the therapeutic effect remains insufficient [11]. Recently, we constructed a novel adenovirus 5-based Ad-TD, which had three gene deletions (*E1ACR2*, *E1B19K* and *E3gp19K*) but retains the *E3B* gene. This new mutant oncolytic adenovirus exhibited strong anti-tumor efficacy in vivo [12]. On the basis of Ad-TD, we constructed a novel oncolytic adenovirus Ad-TD-nsIL12, armed with human non-secretory interleukin-12 (ns*IL12*, deletion of the signal peptide). IL-12 is considered to be an attractive antitumor molecule by activating anti-tumor immunity and inhibiting tumor angiogenesis [13]. However, systemic administration of IL-12 classically results in the development of intolerable adverse events. To overcome this, delivery of IL-12 using OAd localizes expression of IL-12 within the tumor microenvironment (TME) [14]. Additionally, use of a non-secreting form of IL-12 further controls the local levels of IL-12, limiting expression within the TME and preventing systemic toxicity. Our previous studies showed that Ad-TD-nsIL12 was more potent compared to unarmed Ad-TD in Syrian hamster pancreatic cancer models, and the safety was significantly improved compared to that of Ad-TD-IL12 [12]. 

In order to evaluate the efficacy of Ad-TD-nsIL12 in ESCC, the selection of the evaluation model is critical. Although cancer cell lines are widely used to study the molecular mechanisms associated with cancer and evaluate therapeutic drug responses, emerging evidence demonstrates that these widely used cell lines cannot truly mimic the heterogeneity of tumors [15]. Primary patient-derived tumor cells (PDCs) maintain the biological and genomic characteristics of original tumors well, and have a high rate of tumorigenesis, which make them a more precise system for drug evaluation [16]. Inoculation of human tumor cells subcutaneously into immune-deficient mice is commonly used for the establishment of human tumor models in vivo. However, these models do not faithfully reflect the histological features of the primary tumor. Our recent research revealed that immune-deficient Syrian hamster model could be used to more accurately recapitulate histological features and pathological progression of human tumors [17]. In addition, human Ads replicate very poorly in mice, but can replicate well in the Syrian hamster model [18]. Thus, the immune-deficient Syrian hamster model is an ideal animal model for primary evaluation of the effect of OAds in human tumors.

In this study, we compared the therapeutic effect of Ad-TD-nsIL12, H101 and traditional cisplatin treatment using primary patient-derived tumor cells and a panel of ESCC cell lines in immune-deficient Syrian hamsters and provide a new strategy for the treatment of esophageal cancer.

## 2. Materials and Methods

### 2.1. Ethics Statement 

Informed written consent was obtained from the patients. Ethical approval was obtained from the Ethics Committee of Zhengzhou University (2016/Wang). Sample collection conformed to the provisions of the Declaration of Helsinki. 

### 2.2. Cell Culture and Cell Lines 

Fresh tumor tissues were obtained from ESCC patients. Samples were diced, digested with digestive juice containing 1 mg/mL collagenase type I (Sigma-Aldrich, S.t. Louis, MO, USA), 1 mg/mL collagenase type IV (Sigma-Aldrich), filtered, then resuspended in Dulbecco’s Modified Eagle Medium (DMEM)/F-12 Medium (Gibco, Carlsbad, CA, USA), supplemented with 5% Fetal Bovine Serum (FBS, Gibco). The culture medium was changed every 3 days. Fibroblasts were removed by incubation with 0.05%Trypsin/EDTA. The primary patient-derived tumor cells were named SBRC-EC01 and SBRC-EC02. For the experiments described here, all cells were used in passages 10 to 16. The cell lines KYSE510, KYSE180, KYSE270 and other ESCC cell lines were obtained from the Japanese Collection of Research Bioresources Cell Bank (JCRB, Osaka, Japan), and passaged in Roswell Park Memorial Institute-1640 (RPMI 1640, Gibco) with 10% FBS. The human kidney epithelial cell line HEK-293 was purchased from the Cell Bank of the Type Culture Collection Committee of the Chinese Academy of Sciences (Shanghai, China) and maintained in DMEM supplemented with 10% FBS. All cells were verified free of mycoplasma and bacterial contamination.

### 2.3. Viruses and Agents

Recombinant adenovirus H101 (human adenovirus type 5 with deletions of the *E1B55K* and *E3B* genes) was purchased from Shanghai Sunway Biotech Co., Ltd. (Shanghai, China). Recombinant adenovirus 5-based Ad-TD-LUC with *E1ACR2*, *E1B19K*, *E3gp19K*-triple deletions and luciferase insertion, and Ad-TD-nsIL12 with *E1ACR2*, *E1B19K*, *E3gp19K*-triple deletions and non-secreted IL-12 (nsIL12, without signal peptide fragments of IL-12) insertion were constructed in our laboratory as previsously described [12]. Ad-TD-LUC and Ad-TD-nsIL12 were purified by CsCl gradient centrifugation. The virus physical partical titre was measured using optical absorbance (OD260) of disrupted virions and the infectious titer was measured by the 50% tissue culture infection dose (TCID50) method as described previously [12]. The ration of particle and infectious titers for Ad-TD-nsIL12 was 51:1 (5 × 10^11^ VP/mL/9.75 × 10^9^ pfu/mL), for Ad-TD-LUC was 4.5:1 (11 × 10^11^ VP/mL/2.44 × 10^11^ pfu/mL), and for H101 was 86:1 (10 × 10^11^ VP/mL /1.16 × 10^10^ pfu/mL). Anti-cancer drugs cisplatin and 5-fluorouracil (Selleckchem, Houston, TX, USA) were used. The stock solution of cisplatin was reconstituted with Phosphate Buffered Saline (PBS, Sigma-Aldrich), and the stock solution of 5-fluorouracil was reconstituted with DMSO.

### 2.4. Cell Proliferation Assay

5 × 10^4^ cells at passage 15 were seeded in 24-well plates. The cell numbers were counted every 24 h for 7 days. The growth curves were plotted and the doubling time at the exponential growth phase was calculated [19]. Each point shows mean ± SD (*n* = 3).

### 2.5. Colony Formation Assay

600 cells were seeded into 100 mm culture plates, and incubated at 37 °C with 5% CO_2_ for 2 weeks. The cell culture medium was changed every 3 days. After washing with PBS, cells were fixed with 4% paraformaldehyde for 5 min, then stained with 1% crystal violet for 10 min. Only colonies with cells >50 were counted. Colonies were examined and calculated. The data were expressed as mean ± SD (*n* = 3).

### 2.6. Cell Viability Assays

For the chemotherapeutic drug cytotoxicity assay, cancer cells were seeded in 96-well plates at 4000 cells/well, and cultured in DMEM with 10% FBS for 24 h, then treated with various concentrations of drugs for 72 h in a 37 °C incubator with 5% CO_2_. Cell viability was examined using the MTS assay (Promega, Madison, WI, USA). The IC50 value (half maximal inhibitory concentration) was calculated. Experiments were performed three times using cells at different passage numbers. Cell viability in each well was calculated according to the following formula: Cell viability = (absorbance value of treated cells − background)/(absorbance value of untreated control cells − background), and expressed as a percentage of that for untreated cells [20].

For the virus cytotoxicity assay, cancer cells were seeded in 96-well plates at 2500 cells/well in DMEM with 2% FBS for 18 h, then infected with viruses at a starting multiplicity of infection (MOI) of 1000 plaque forming units (PFU)/cell. Cell viability was determined by MTS assay 6 days later, and EC50 values (viral dose killing 50% of tumor cells) were calculated [10]. All data presented were from three independent infection studies.

### 2.7. Viral Replication Assay

To evaluate viral replication in human ESCC cells, tumor cells were seeded in 6-well plates at 2 × 10^5^ cells/well in 2 mL DMEM with 10% FBS, then incubated at 37 °C, 5% CO_2_. 18 h later, cells were infected with 5 PFU/cell of virus. Samples were collected in triplicate at 24 h, 48 h, 72 h and 96 h after infection. The samples were titered on HKE293 cells to determine the 50% tissue culture infective dose [10].

### 2.8. ELISA 

IL-12 was determined as described previously [12]. Briefly, cancer cells were infected with Ad-TD-nsIL12. Supernatant and lysate were collected after 24 h, 48 h, 72 h and 96 h. IL-12 levels were quantified using human IL-12 p70 ELISA (eBioscience, San Diego, CA, USA) in triplicate according to the manufacturer’s protocol.

### 2.9. In Vivo Animal Studies

All the animal experiments in this study were approved by the Animal Welfare and Research Ethics Committee of Zhengzhou University (Zhengzhou, China) and were carried out in accordance with the Provision and General Recommendation of Chinese Experimental Animals Administration Legislation. All the animals were maintained in a laminar airflow cabinet under specific pathogen-free, 12 h dark-light cycle conditions.

Three types of immune-deficient animal models, female B-NDG mice (NOD*-Prkdc^scid^ IL2rg^tm1^*, Biocytogen, Beijing, China), female BALB/c Nude mice (Beijing Vital River Laboratory, Beijing, China) and male ZZU001 hamster were selected for the comparison of tumorigenesis experiment. The transgenic immune-deficient Syrian hamster ZZU001 (*IL2RG^−/−^*) was independently developed by our laboratory platform [17]. 1 × 10^7^ SBRC-EC01 cells were resuspended in 150 µL PBS, then implanted subcutaneously into the right flanks of 5-week to 6-week-old experimental animals. The length and width of tumors were measured by calipers twice a week. The volume of the tumor was calculated using the following formula: volume = (length × width^2^ × π)/6.

To determine the anti-cancer effect of Ad-TD-nsIL12, H101 and cisplatin in subcutaneous transplantation tumor models of SBRC-EC01 in vivo, ZZU001 hamsters were inoculated subcutaneously into the right flanks with 1 × 10^7^ cancer cells. When tumors reached 300 mm^3^, the tumor-bearing animals were divided into four groups: PBS group, Ad-TD-nsIL12 group, H101 group and cisplatin group (*n* = 7/group). One hundred µL PBS or Ad-TD-nsIL12 (5 × 10^8^ PFU/hamster) or H101 (5 × 10^8^ PFU/hamster) in 100 µL PBS were injected intratumorally on day 0, 2, 4, 6, 8 and 10. Cisplatin (3 mg/kg) was injected intraperitoneally once a week for 4 weeks. Tumor volumes were estimated using electronic calipers [tumor volume = (length × width^2^ × *π*)/6] twice a week. When tumors reached 3500 mm^3^ or tumor ulceration occurred or animals lost 20 percent of their body weight, hamsters were sacrificed, and organs were harvested to investigate histopathological characteristic and distant metastasis. 

### 2.10. Histopathological Examination and Immunohistochemistry

Tumor cells growing on slides were washed with PBS and fixed in 4% paraformaldehyde for 10 min [21]. Tumor tissues from the patients and subcutaneous tumors were fixed in 4% paraformaldehyde, embedded in paraffin, and sectioned at 6 μm. Tumor cells and sections were stained with hematoxylin-eosin (H&E). For immunohistochemistry (IHC), tumor sections were repaired in antigen repair solution for 10 min. Tumor sections and tumor cell slides were blocked with 3% H_2_O_2_ for 10 min, washed with PBS and blocked with 10% goat serum for 30 min, then incubated overnight with primary antibodies. The slides were washed with PBS and followed by incubation with a secondary antibody. Primary antibodies were as follows: anti-E1A (1:100 dilution, Clone M58, GeneTex, San Antonio, TX, USA), anti-Ki67 (Clone OTI8H5, ZXGB-BIO, Beijing, China), anti-p63 (Clone 4A4+UMAB4, ZXGB-BIO, Beijing, China), Pan Cytokeratin (AE1/AE3) (Clone MX005, MXB Biotechnologies, Fuzhou, CHN), and anti-CD31 (1:1000 dilution, Clone ab182981, Abcam, Cambridge, MA, USA). The slides were imaged by a BX41 microscope (Olympus, Tokyo, Japan).

Samples were cut in at least 3 sections at different time points. Positive cells were counted in five randomly selected high-power fields (200×) per slide. Percentages of positive cells were analyzed using Image-pro-plus software. For CD31, positive cells were counted using a light microscope and any endothelial cell or cluster of endothelial cells separated from other neighboring clusters of endothelial cells was counted as a unit of blood vessel cluster [22].

### 2.11. Statistical Analysis

All data presented were from at least 3 separate experiments and are shown as mean ± SD. Statistical analysis was performed using Graph Pad Prism 5 (La Jolla, CA, USA). Differences were evaluated using one or two-way ANOVA or Student’s *t*-test or Kaplan–Meier survival analysis. A significance level of *p* < 0.05 was regarded as statistically significant.

## 3. Results

### 3.1. Establishment of Two ESCC Patient-Derived Tumor Cells (PDCs) 

**Figure 1:**
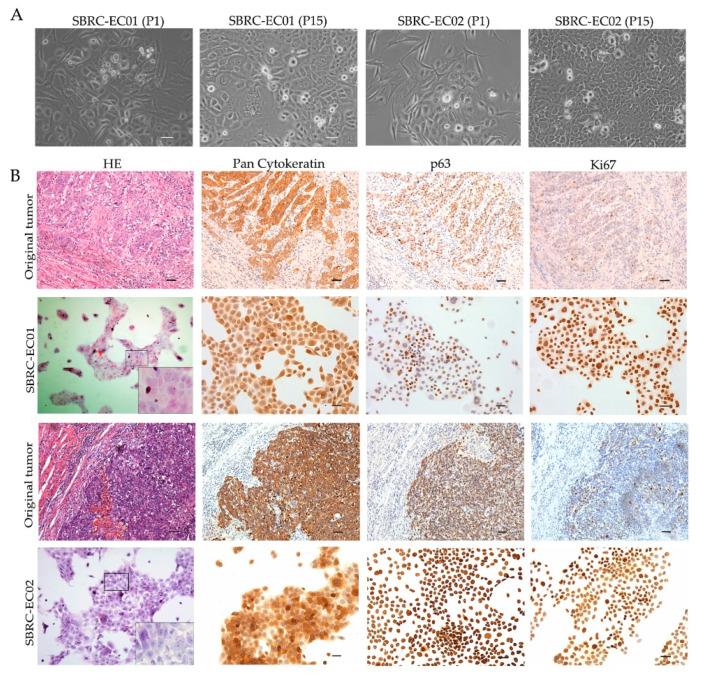
SBRC-EC01 and SBRC-EC02 mimic the biological characteristics of the primary tumors. (**A**) Phase contrast images of SBRC-EC01 and SBRC-EC02 at different passages are presented. Polygonal epithelial-like cells were surrounded by long fusiform cells at passage 1. At passage 15, cells of different shapes and sizes were observed; (**B**) Histopathological examination of the original tumors and the derived cell populations. Immunostaining for Pan Cytokeratin (AE1/AE3), Ki67, p63 are shown. Scale bar: 50 μm.

To establish an accurate model for evaluating drug responses, patient-derived tumor cells named SBRC-EC01 and SBRC-EC02 were created using fresh tumor tissues from ESCC patients. The cells grew as a monolayer, with variable shapes and sizes, which reflected the heterogeneity seen within the tumor (Figure 1A). The cells showed a strong proliferative capacity, colony forming abilities and migration abilities (Figure S1). IHC was performed on the original tumors and the derived cell populations. The epithelial origin marker Pan Cytokeratin (AE1/AE3) showed strong positivity in both the original tumors and the derived cell populations. As measured by the proliferation marker Ki67 staining, cell proliferation was lower for the original tumors (20 ~ 40%), but high for the derived cells (100%). The keratinocyte proliferation marker p63 showed strong positivity in tumor-derived cells and the original tumors (Figure 1B). STR analysis proved the uniqueness of the cells, and there was no cross-contamination with other cells (Table S1). The results demonstrated that the newly established tumor cells maintained similar morphology, molecular biology and heterogeneity as the original tumors. 

### 3.2. Effect of Conventional Chemotherapy Drugs in ESCC Cell Lines and the Two PDCs In Vitro

**Figure 2:**
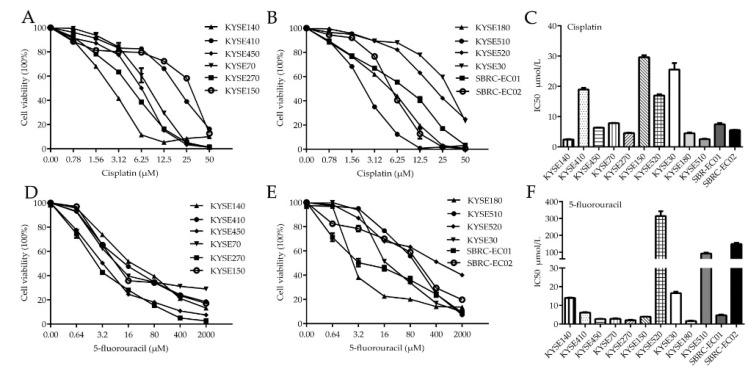
Cell Viability assays display the effect of 5-fluorouracil and cisplatin in ESCC cell lines and two patient-derived tumor cells. (**A**–**C**) Cytotoxicity assay of cisplatin in ESCC cell lines and two patient-derived tumor cells. (**D**–**F**) Cytotoxicity assay of 5-fluorouracil in ESCC cell lines and two patient-derived tumor cells. Patient-derived tumor cells include SBRC-EC01 and SBRC-EC02. Tumor cells were treated with increasing concentrations of drugs for 72 h. Cell viability was measured using MTS assay, and IC50 value (half maximal inhibitory concentration) was calculated. Data are shown as the mean ± SD of 3 independent experiments.

For ESCC, the most effective treatment involves a combination of fluorouracil and cisplatin, sometimes in combination with a third drug such as tislelizumab [23]. The patients from whom SBRC-EC01 and SBRC-EC02 were derived also received adjuvant fluorouracil and platinum chemotherapy after surgical debulking. In order to assess the effectiveness of chemotherapy drugs and thus guide the clinical treatment of the patients, cisplatin and 5-fluorouracil were investigated using cell viability assays. Among the ten detected cells lines and two PDCs, four cell lines including KYSE150, KYSE30, KYSE520 and KYSE410 showed a substantially higher tolerance to cisplatin with the IC50 of (29.6 ± 0.98 μmol/L), (25.54 ± 3.03 μmol/L), (16.98 ± 0.55 μmol/L) and (18.94 ± 0.76 μmol/L) respectively, which were comparable to previously reported IC50 values of ESCC cells for cisplatin (3.39 ± 0.087 μmol/L) (Table S2) [20]. SBRC-EC01 and SBRC-EC02 were sensitive to cisplatin with the IC50 of (7.48 ± 0.55 μmol/L) and (5.55 ± 0.14 μmol/L) respectively. Two cell lines including KYSE520, KYSE510 and SBRC-EC02 showed a higher tolerance to 5-fluorouracil with the IC50 of (314.63 ± 40.86 μmol/L), (92.08 ± 7.64 μmol/L) and (148.47 ± 9.18 μmol/L) respectively, which were comparable to previously reported IC50 values of ESCC cells for 5-fluorouracil (78.57 ± 2.94 μmol/L) (Table S2) [20]. Although SBRC-EC01 was sensitive to 5-fluorouracil, there was a dose-related plateau in their ability to kill cancer cells (Figure 2). These results suggest a resistance to conventional chemotherapy in the detected cell lines and newly established PDCs.

### 3.3. Cytotoxicity and Replicating Ability of Ad-TD-nsIL12, Ad-TD-LUC and H101 in ESCC Cell Lines and the Two PDCs In Vitro

**Figure 3:**
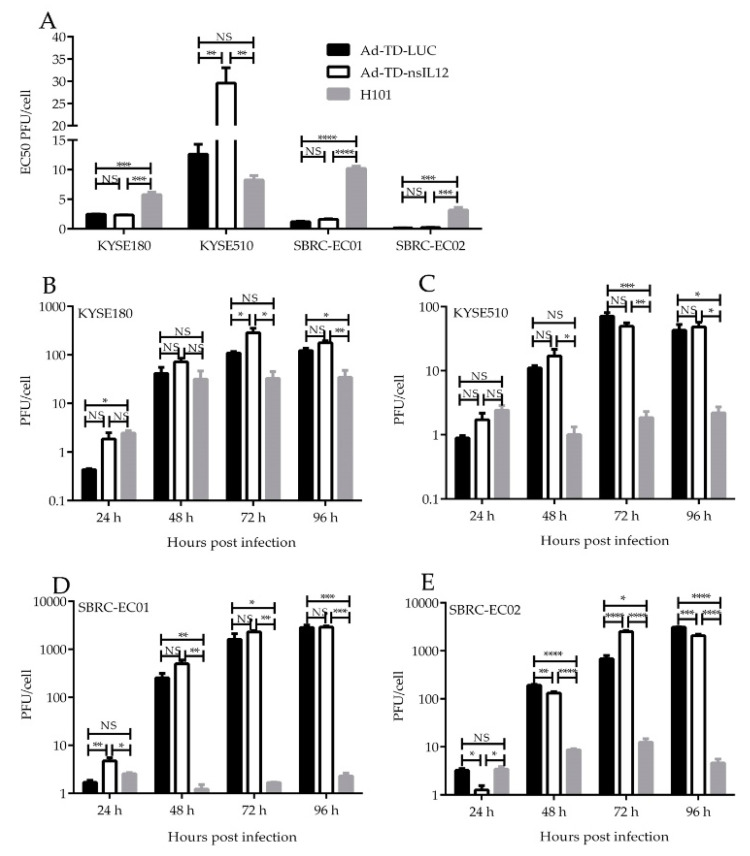
AD-TD-LUC, Ad-TD-nsIL12 and H101 can replicate in and cause oncolysis of human ESCC cell lines and PDCs. (**A**) To assess virus cytotoxicity, cell proliferation assays were carried out in two PDCs and two ESCC cell lines (KYSE180, KYSE510). Cells were infected with AD-TD-LUC, Ad-TD-nsIL12 or H101 separately. The cell viability was determined by the MTS assay 6 d after infection. EC50 value for each virus is shown as a measure of the cytopathic effect; (**B**–**E**) Replication assays were carried out in two ESCC cell lines (KYSE180, KYSE510), SBRC-EC01 and SBRC-EC02 at a MOI of 5 PFU/cell. Data are expressed as mean ± SD (*n* = 6) and analyzed by one way ANOVA. * *p* < 0.05, ** *p*< 0.01, *** *p* < 0.001, **** *p* < 0.0001. NS, no statistical significance.

To characterize the cytotoxicity of OAds, the cytotoxic effect and replication ability of Ad-TD-nsIL12, control virus Ad-TD-LUC and H101 were assessed in SBRC-EC01, SBRC-EC02 and a panel of ESCC cell lines in vitro. Ad-TD-nsIL12 exhibited a better cytotoxic effect on the majority of human ESCC cell lines and the PDCs than Ad-TD-LUC and H101. Two cell lines, KYSE510 and KYSE150 showed some resistance to Ad-TD-nsIL12 (Figure 3A, Figure S2). 

Ad-TD-nsIL12 showed better replication capability than H101, which replicated poorly in the tumor cells lines tested (Figure 3B–E). There was no significant difference in the replication ability of Ad-TD-nsIL12 and Ad-TD-LUC in most cell lines. 

The amount of human IL-12 produced after infection of ESCC cell lines and PDCs with Ad-TD-nsIL12 was examined using ELISA to quantify IL-12 in cell culture supernatant and cell lysate. In the early stage of Ad-TD-nsIL12 infection (within 24 h), low levels of IL-12 was detected from supernatants and lysate, which steadily increase from 48 h post infection, but peaked at a maximum level of not more than 6 ng/mL in the supernatant, demonstrating that each cell line supported viral replication and transgene expression, but nsIL-12 was not released in significant amounts from intact cells (Figure S3). Of note, both supernatant and cell lysates presented the similar level of IL-12 allthough it is expected that IL-12 expression in supernatant should be lower than those in cell lysates. This might be due to that the four cell lines were very infectable to adenovirus and Ad-TD-nsIL12 replicated rapidly and lysed these cells even at 24 h after the virus infection. 

### 3.4. Anti-Tumor Effects of Ad-TD-nsIL-12, H101 and Cisplatin in SBRC-EC01 Subcutaneous Xenograft Tumor Model 

Research has shown that human IL-12 is non-functional in murine systems, but can work within the Syrian hamster immune system [12]. Hamster cells can support adenovirus replication, thus providing a strong model for evaluation of efficacy of OAds [24]. In addition, SBRC-EC01 had strong tumorigenicity in the immune-deficient Syrian hamster (ZZU001), but not in B-NDG mice or BALB/c Nude mice, and the pathological structure of subcutaneous tumor was similar to the primary tumor (Figure S4). Therefore, the ZZU001 hamster model was used to evaluate the therapeutic effect of Ad-TD-nsIL12. 

**Figure 4:**
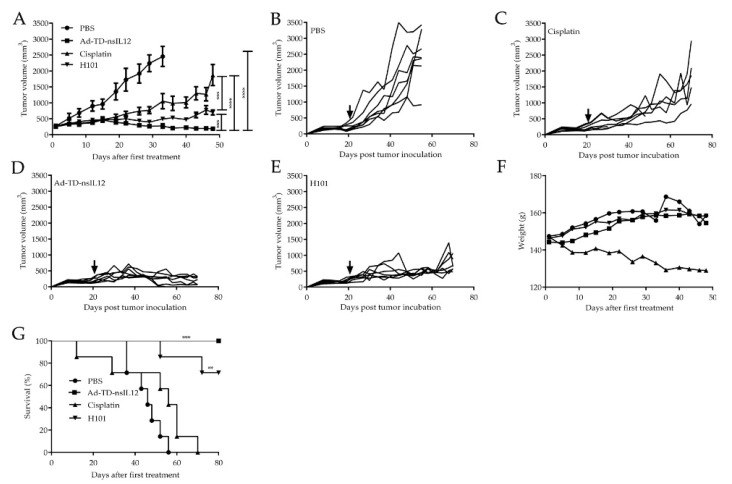
Ad-TD-nsIL12 can effectively control tumor growth in SBRC-EC01 subcutaneous xenograft tumor model. (**A**–**E**) SBRC-EC01 cells were used to establish a xenograft model in ZZU001 hamsters. 1 × 10^7^ cells were seeded into the right flank of ZZU001. When the tumors reached 300 mm^3^, Ad-TD-nsIL12 (5 × 10^8^ PFU), H101 (5 × 10^8^ PFU) or PBS were each injected intratumorally on days 0, 2, 4. 6, 8 and 10; 3 mg/kg cisplatin was intraperitonealy injected once a week for 4 weeks. Ad-TD-nsIL12 could significantly inhibit the tumor growth compared with other groups. The arrow shows the start time of treatment. Tumor sizes were expressed as the mean ± SD in each group. Statistical significance was determined using a two-way ANOVA and a Student’s *t*-test for the comparison between groups; (**F**) The body weight of virus treatment group and control group increased slowly. But in cisplatin group, the body weight of all hamsters decreased; (**G**) Kaplan–Meier survival curves were generated and significance was assessed using the log-rank (Mantel–Cox) test. ** *p* < 0.01, *** *p* < 0.001, **** *p* < 0.0001.

To determine whether Ad-TD-nsIL12 can inhibit the growth of tumors in vivo, a subcutaneous SBRC-EC01 xenograft tumor model was established in ZZU001 hamsters. Subcutaneous SBRC-EC01 (1 × 10^7^ cells) tumors were established in the flank of male ZZU001 hamsters and treated when the tumor volume reached 300 mm^3^. Animals were each injected intratumorally with Ad-TD-nsIL12 (5 × 10^8^ PFU) or H101 (5 × 10^8^ PFU) or PBS six times. For the cisplatin treatment group, animals were each injected intraperitoneally with 3 mg/kg cisplatin once a week for 4 weeks. Compared with the PBS group, Ad-TD-nsIL12 had a significant therapeutic effect on subcutaneous xenograft tumors (*p* < 0.0001). One of seven animals in the group was cured and remained tumor-free for more than 30 days (Figure 4A,B,D). The animals were in good condition without weight loss (Figure 4F) and no animals in this treatment group died prior to the experimental endpoint (Figure 4G). Compared with the Ad-TD-nsIL12 group, the treatment effect of cisplatin was poor (*p* < 0.0001). Cisplatin could significantly inhibit the growth of tumor within 4 weeks, but tumor recurrence occurred before the experimental endpoint. In addition, cisplatin induced a loss in body weight and caused mortality after treatment compared with the virus treatment group (Figure 4A,C,F,G). Compared with the Ad-TD-nsIL12 group, the therapeutic effect of H101 was worse (*p* < 0.001). Although H101 could significantly inhibit the growth of tumor, the tumor did not disappear (Figure 4A,E). Of note, the PBS group had distant metastasis (2/7), but none of the treatment groups had metastasis, indicating that the treatment methods adopted can effectively inhibit tumor metastasis (Figure S5).

### 3.5. Ad-TD-nsIL12 Demonstrates Prolonged Gene Expression in Tumor Tissues, Inhibits the Proliferation of Tumor Cells and Reduces the Microvessel Density of Tumor Tissue in ZZU001 Hamster Models

**Figure 5:**
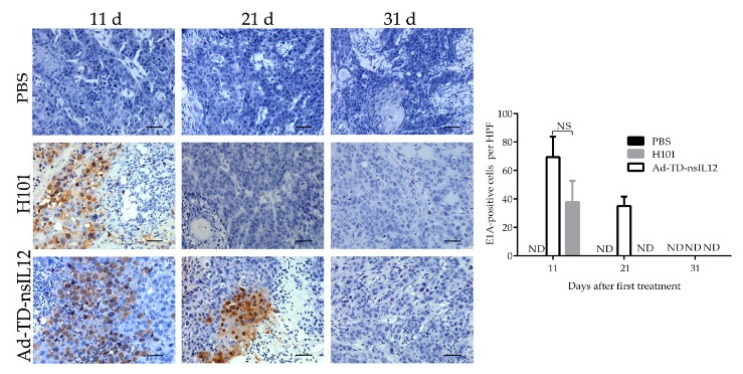
Ad-TD-nsIL12 persists in tumor tissue for a longer duration than H101. Tumors growing in ZZU001 were directly injected with Ad-TD-nsIL12 or H101 (5 × 10^8^ PFU) on days 0, 2, 4, 6, 8, and 10. On days 11, 21 and 31 after the first viral injection, three tumors harvested from each treatment group were analyzed for expression of E1A. The data are calculated as mean ± SD and analyzed by one way ANOVA. ND, undetectable.

We compared the viral early protein E1A expression after intratumoral injection of Ad-TD-nsIL12 or H101 in immune-deficient Syrian hamsters by IHC. On the 11th day after the first viral injection, high levels of E1A were detected in the tumor tissues of the Ad-TD-nsIL12 and H101 treatment groups. On the 21st day, a high level of E1A was still detected in the tumor tissues of the Ad-TD-nsIL12 animals, but none was detected in H101-treated animals at this timepoint. No viral protein was detected in either treatment group by day 31 post-infection (Figure 5).

**Figure 6:**
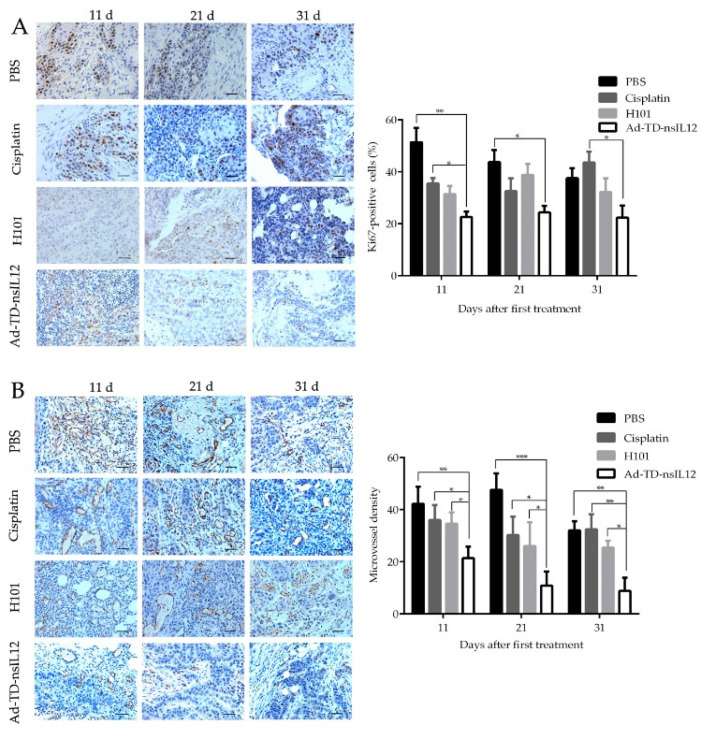
Ad-TD-nsIL12 can inhibit the proliferation activity of tumor cells and reduce the microvessel density of tumor tissue. (**A**) Ki67 staining of tumor cells in xenografts. The percentage of Ki67 positive cells was determined in five high-power fields of tumor section; (**B**) CD31 staining of xenografts in each group. The mean microvascular density was determined by counting CD31-positive microvascular structures in five high-power fields of tumor section. The data are calculated as mean ± SD and analyzed by one way ANOVA. Scale bar: 50 μm. * *p* < 0.05, ** *p* < 0.01, *** *p* < 0.001.

The proliferation marker Ki67 is a promising target for cancer therapy and can predict cancer progression independently [25]. The proliferation activity of tumor tissue was evaluated by IHC using the Ki67 antibody. Five fields were randomly selected under 200× microscope, and the percentage of positive cells was calculated. Compared with cisplatin or PBS groups, the percentage of Ki67-positive tumor cells in the Ad-TD-nsIL12 group was significantly decreased at all timepoints (*p* < 0.05) (Figure 6A), demonstrating that Ad-TD-nsIL12 could effectively inhibit the proliferation of tumor cells. Conversely, cisplatin had the weakest anti-prolifertive effect.

The number of microvessels including capillaries and venules in tumor tissue was determined by anti-CD31 labelling. 11 days after the first treatment, a marked reduction of vascularity was observed in the Ad-TD-nsIL12 group (*p* < 0.01), which might be related to the previously defined anti-angiogenic effect of IL-12 [26]. Coarse and irregular networks of microvessels were found in other groups (Figure 6B). 

## 4. Discussion

In recent years, oncolytic viruses (OVs) have emerged as a novel anticancer treatment [27]. Adenoviruses are the most commonly used viral vectors for gene therapeutic applications [28]. Oncolytic adenovirus was the first approved OV therapy for clinical use [9]. H101, the first-generation oncolytic adenovirus has both the *E1B55K* and the *E3B* genes deleted and was approved in China for treatment of head and neck cancer [9]. However, given that a major function of E1B55K protein is to promote the nuclear export of the late viral mRNAs to the cytoplasm for viral protein synthesis, deletion of *E1B55K* affects virus replication, attenuating the potential anti-tumor effect associated with H101 [29,30]. Deletion of the *E3B* gene region also attenuates viral replication and accelerated virus clearance, thus further reducing antitumor efficacy [10]. The new generation OAd, Ad-TD-LUC, has a triple gene deletion (*E1ACR2*/*E1B19K*/*E3gp19K*), but retains the *E1B55K* and *E3B* gene function, preventing replication attenuation in tumor cells [12]. Deletion of *E1B19K* and *E3gp19K* increases tumor selectivity of the virus [31]. Further, the *E3gp19K* deletion increases virus replication, cytotoxic T lymphocyte infiltration and antitumor efficacy associated with OAd [10]. Efficacy of OAds can be improved through co-expression of therapeutic genes [32]. Ad-TD-nsIL12 is based on the Ad-TD-LUC backbone, but includes an expression cassette for a modified human non-secreting IL-12 (ns*IL12*) gene in the *E3gp19K* region [12]. IL-12, a powerful immunoregulatory factor, exerts anti-tumor effects by activating dendritic cells, macrophages, NK cells and cytotoxic T lymphocytes. It promotes these cells to produce INF-γ, resulting in the development of anti-tumor immunity and the inhibition of tumor angiogenesis [33,34]. Systemic administration of IL-12 has been evaluated previously, but was found to produce a significant toxic effect, greatly hindering its clinical application [35]. OAds delivering IL-12 were tested in phase I clinical trials for pancreatic cancer, glioblastomas and oligoastrocytomas. However, the virus resulted in release of high levels of IL-12 into serum, which resulted in severe toxicity in patients [28,36,37]. Ad-TD-nsIL12 expresses human non-secretory *IL12*. With the replication of virus restricted to tumor cells, IL-12 produced by the virus accumulates in the cytoplasm of infected cells. nsIL-12 is released slowly and controllably by lysis of tumor cells, rather than active secretion, thus controlling the localization of IL-12 expression within the tumor microenvironment. Using this transgene, only a slight increase of IL-12 in peripheral blood was noted previously and Ad-TD-nsIL12 was shown to have potent antitumor effects, with no systemic toxic side-effects in vivo [12].

In order to evaluate the therapeutic effect of Ad-TD-nsIL12 in ESCC, we established two PDCs from human tumor samples of ESCC, named SBRC-EC01 and SBRC-EC02. Research has indicated that PDCs, especially earlier generations of cells are more heterogeneous and have more similar characteristics to primary tumor [38]. Our results showed the two PDCs retained the molecular characteristics of the original tumors, and were valuable models to evaluate the therapeutic efficacy of anti-tumor drugs.

Using the two newly established PDCs and a panel of conventional ESCC cell lines, we first evaluated the effectiveness of chemotherapeutic drugs. The patients from whom the PDCs were derived were treated with platinum and fluorouracil as the first-line chemotherapy, so chemotherapeutic drugs such as cisplatin and 5-fluorouracil were used to kill the cells in vitro. SBRC-EC01 was relatively sensitive to cisplatin and although it was also reasonably sensitive to 5-fluorouracil, there was a drug treatment plateau. SBRC-EC02 was sensitive to cisplatin, but resistant to 5-fluorouracil treatment. In addition to drug resistance, chemotherapy often produces adverse reactions such as neutropenia, myelosuppression, and even promotion of tumor metastasis [39]. Therefore, more effective and less toxic drugs are urgently needed to replace conventional chemotherapy drugs.

We investigated the cytotoxic activity of oncolytic adenovirus H101, Ad-TD-LUC and Ad-TD-nsIL12 in ESCC cell lines and the two newly established PDCs. The three viruses were cytotoxic to all cancer cells examined. Higher cytopathic effects were observed for Ad-TD-nsIL12 compared with Ad-TD-LUC and H101 except in KYSE510 and KYSE150. Viruses could replicate in all the tumor cells and Ad-TD-nsIL12 had stronger replication ability compared to H101. The replication ability of H101 was poor except in KYSE180. After infection of Ad-TD-nsIL12, only low levels of IL-12 were detected in the cell supernatant. The results showed that Ad-TD-nsIL12 had better cytopathic effect and replication capability in the majority of cell lines and the two PDCs. It could controllably release IL-12 to cell supernatant after lytic infection of tumor cells.

Having confirmed the efficacy of Ad-TD-nsIL12 in vitro, we next investigated the antitumor efficacy in vivo. For efficacy evaluation of Ad-TD-nsIL12, the selection of animal models is particularly important. Syrian hamster are permissive for human Ad replication, however mouse models are not [40]. Therefore, Syrian hamster is considered as a superior model to test the efficacy and safety of OAds [24]. Research has indicated that human IL-12 is non-functional in murine models, whereas it is functional in the Syrian hamster [12]. In addition, SBRC-EC01 had the strongest tumorigenicity in ZZU001, a transgenic Syrian hamster model (*IL2RG^−/−^*). Thus, ZZU-001 is an ideal animal model to evaluate the therapeutic effect of Ad-TD-nsIL12 using a SBRC-EC01 xenograft model.

*In vivo* studies revealed that while all the treatments could effectively inhibit tumor metastasis, the effects of oncolytic viruses were better than that of cisplatin. The difference in control of the primary tumor was more marked, with Ad-TD-nsIL12 outperforming cisplatin and H101. Tumors in cisplatin group started growing rapidly after the last treatment. Cisplatin also caused side effects such as weight loss and increased mortality. Intratumoral inoculation with Ad-TD-nsIL12 markedly inhibited tumor growth and resulted in 100% survival, and the therapeutic effect of Ad-TD-nsIL12 was better than that of H101. However, the ZZU001 model has a severe immunodeficiency phenotype, lacking mature T, B and NK cells, and hence the immune promoting effect of IL-12 could not be evaluated effectively in this model. This suggests that the therapeutic effect of Ad-TD-nsIL12 on the tumor has not been fully exerted and in the presence of an intact immune system, the therapeutic effect is expected to be even stronger that we have shown here. Of note, there is currently no any Syrian hamster-derived esophageal cancer cell line or in vivo model available for evaluating the antitumour efficacy and safety of Ad-TD-nsIL12 and other immunotherapeutic agent for treatment of esophageal cancer.

Consistent with the previous studies, regardless of immune status of animals, the virus in tumor tissues began to decline and eventually disappeared after the treatment [41]. We further examined the oncolytic Ad E1A protein expression in tumor tissue at the indicated time points. Ten days after the first viral injection (one day after the last treatment), highly expressed early viral protein E1A could be detected in tumor tissues of both groups. 21 days after the first viral injection, E1A protein expression was still detected in the Ad-TD-nsIL12 group, but not in the H101 group. On day 31 after the first viral injection, there was no detectable E1A protein expression in tumors of both groups. Thus, H101 has a weaker abililty to replicate and/or a faster clearance rate compared to Ad-TD-nsIL12, as suggested by the functional losses as a result of the *E1B55K* and *E3B* gene deletions in H101. CD31 expression in blood vessels is a very important marker for detection of tumor angiogenesis. Tumors in the Ad-TD-nsIL12 group showed weaker CD31 expression compared to the other groups. The result suggested that Ad-TD-nsIL12 could inhibit angiogenesis of tumor tissues by generating IL-12. Both Ad-TD-nsIL12 and H101 could inhibit the proliferation of SBRC-EC01 cells in tumors more strongly than cisplatin. Cisplatin could inhibit the growth of tumor cells in the early stage of treatment, but the proliferation of the remaining tumor cells was significantly enhanced subsequently. This led to a rapid recurrence of the tumor. Research suggests that chemotherapy can induce senescence in tumor cells. The senescence condition promotes cancer stemness, which ultimately enhances tumor proliferation and tumor initiation capacity [42]. SBRC-EC01 had obvious tumor heterogeneity. Within the heterogenous tumor cell population, chemotherapy screened out the resistant subpopulations, which eventually caused the proliferation of residual tumor cells and promoted recurrence [43].

In conclusion, Ad-TD-nsIL12 can effectively infect and replicate in human ESCC cells, produce cytotoxic effect, and has better therapeutic effect than H101 and conventional chemotherapy in ESCC subcutaneous transplantation tumors, with no associated toxicity. As such Ad-TD-nsIL12 is a promising candidate anticancer agent for ESCC.

## Supplementary Materials

The following are available online at https://www.mdpi.com/2073-4409/9/11/2438/s1, Figure S1: Growth curves and clone formation rates of SBRC-EC01 and SBRC-EC02 cells at passage 15. Figure S2: Cytotoxicity of AD-TD-LUC, Ad-TD-nsIL12 and H101 in a panel of human ESCC cell lines. Figure S3: The production of IL-12 by Ad-TD-nsIL-12 in infected tumor cells. Figure S4: Compared with B-NDG mice and BALB/c Nude mice, SBRC-EC01 has stronger tumorigenicity in immune-deficient Syrian hamster (*IL2RG^−/−^*, named ZZU001). Figure S5: Histological analysis of SBRC-EC01 tumors. Table S1: STR matching analysis of SBRC-EC01 and SBRC-EC02; Table S2: Drug sensitivity in ESCC cell lines and PDCs.
