# Supplementary material for: A Tumor-Targeted Replicating Oncolytic Adenovirus Ad-TD-nsIL12 as a Promising Therapeutic Agent for Human Esophageal Squamous Cell Carcinoma
Source: Cells. 2020 Nov 10;9(11):2438. doi: 10.3390/cells9112438 (PMC7698064; doi:10.3390/cells9112438)
Supplement: Supplementary file 1 [file cells-09-02438-s001.pdf]

cells

Supplementary Materials: Figure S1-S5; Table S1–S2.

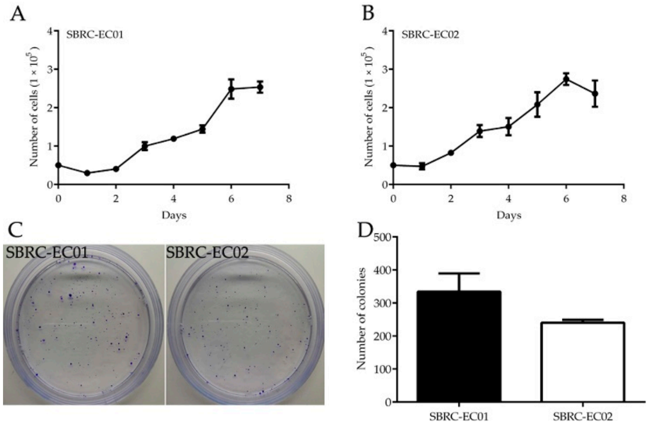

Figure S1. Growth curves and clone formation rates of SBRC-EC01 and SBRC-EC02 cells at passage 15. (A) The growth curve of SBRC-EC01, showing the lag and exponential phase. The population doubling time was 62 h; (B) The growth curve of SBRC-EC02. The population doubling time was 58 h; (C), (D) The clone formation rate was calculated at 2 weeks after 600 single cells seeded into a 100 mm culture plate. Data are shown as the mean ± SD of three independent experiments.

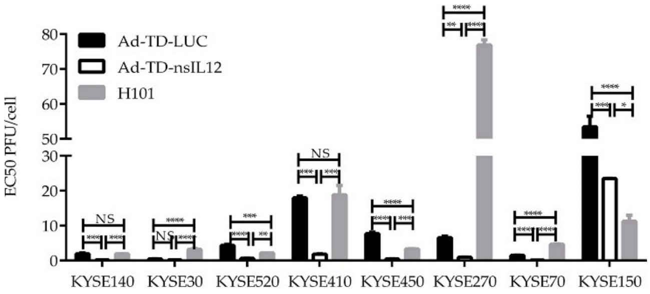

Figure S2. Cytotoxicity of AD-TD-LUC, Ad-TD-nsIL12 and H101 in a panel of human ESCC cell lines. Data are shown as the mean ± SD of three independent experiments and analyzed by one way ANOVA.

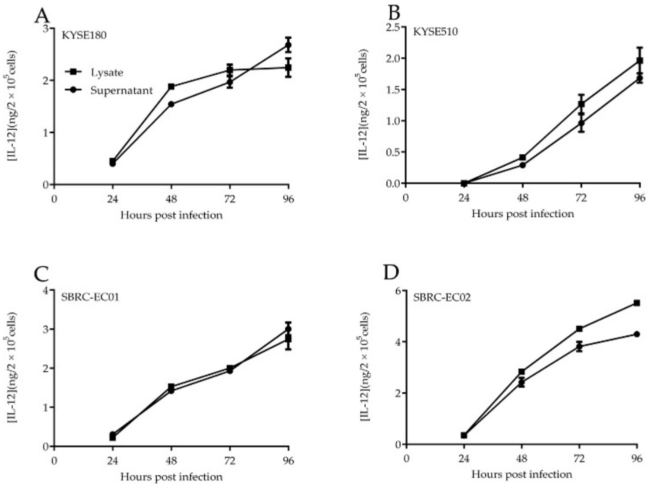

Figure S3. The production of IL-12 by Ad-TD-nsIL-12 in infected tumor cells. To detect IL-12 from Ad-TD- nsIL12 *in vitro*, tumor cells were infected with Ad-TD- nsIL12 at a MOI of 5 PFU/cell. Cell culture

supernatant and lysate were collected every 24 h for 96 h and assayed by ELISA. All experiments were performed in triplicate.

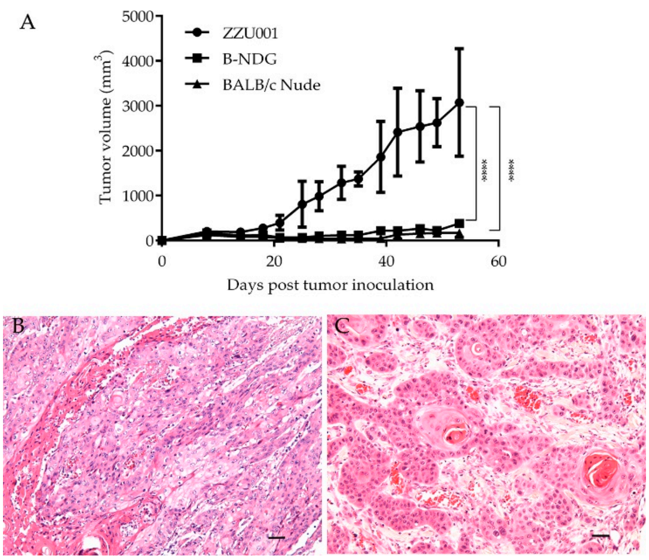

Figure S4. Compared with B-NDG mice and BALB/c Nude mice, SBRC-EC01 has stronger tumorigenicity in immune-deficient Syrian hamster (IL2RG$^{-/-}$, named ZZU001). (A) The tumorigenesis curves of SBRC-EC01 in B-NDG mice, BALB/c Nude mice and ZZU001 hamsters are shown; (B) H&E staining of original tumor; (C) H&E staining of subcutaneous tumor. The tumor volume was measured twice a week. The data are expressed by mean ± SD (n = 3), and analyzed by two way ANOVA. ****, $p < 0.0001$. Scale: 50 µm.

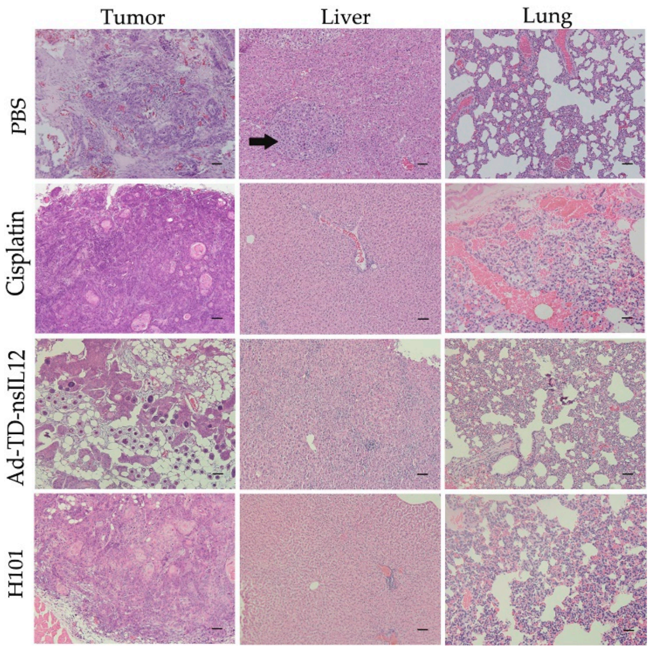

Figure S5. Histological analysis of SBRC-EC01 tumors. Ad-TD-nsIL12 could inhibit the growth and metastasis of tumors. Liver metastasis occurred in PBS group. The arrow shows metastatic deposits.

Table S1. STR matching analysis of SBRC-EC01 and SBRC-EC02.

**Locus**	**SBRC-EC01**	**SBRC-EC02**
D5S818	11,12,13,14	10,10
D13S317	9,12,13	8,8
D7S820	10,11	11,11
D16S539	9,11	11,12
VWA	16,17,19	14,14
TH01	8,8	6,6
AM	X,X	X,X
TPOX	8,11	8,11
CSF1PO	12,15	11,11

Table S2. Drug sensitivity on ESCC cell lines and the two PDCs

	**IC50** (mean ± SD, μM)	
Cell lines	Cisplatin	5-fluorouracil
KYSE150	29.6 ± 0.98	3.9 ± 0.08
KYSE510	2.56 ± 0.19	92.08 ± 7.64
KYSE140	2.46 ± 0.11	13.97 ± 0.28
KYSE410	18.94 ± 0.76	6.15 ± 0.25
KYSE30	25.54 ± 3.03	16.53 ± 1.13
KYSE520	16.98 ± 0.55	314.63 ± 40.86
KYSE450	6.36 ± 0.05	2.72 ± 0.12
KYSE180	4.52 ± 0.39	1.58 ± 0.37
KYSE70	7.84 ± 0.12	2.85 ± 0.31
KYSE270	4.58 ± 0.1	2.04 ± 0.43
SBRC-EC01	7.48 ± 0.55	4.72 ± 0.54
SBRC-EC02	5.55 ± 0.15	148.47 ± 9.18
